# Kinetics of progenitor hemopoetic stem cells in sepsis: Correlation with patients survival?

**DOI:** 10.1186/1471-2334-6-142

**Published:** 2006-09-18

**Authors:** Thomas Tsaganos, Evangelos J Giamarellos-Bourboulis, Spyridon Kollias, Dimitrios Zervakis, Vassiliki Karagianni, Aimilia Pelekanou, Ekaterini-Christina Tampaki, Marina Kontogiorgi, Apostolos Koroneos, Nikolaos Drakoulis, Apostolos Armaganidis, Charis Roussos, Helen Giamarellou

**Affiliations:** 1 4^th ^Department of Internal Medicine, University of Athens, Medical School, Greece; 2 1^st ^Department of Critical Care, University of Athens, Medical School, Greece; 3 Department of Pharmaceutical Technology, University of Athens, School of Pharmacy, Greece; 4 2^nd ^Department of Critical Care, University of Athens, Medical School, Greece

## Abstract

**Background:**

Current theories underline the crucial role of pro-inflammatory mediators produced by monocytes for the pathogenesis of sepsis. Since monocytes derive from progenitor hemopoetic cells, the kinetics of stem cells was studied in peripheral blood of patients with sepsis.

**Methods:**

Blood was sampled from 44 patients with septic syndrome due to ventilator-associated pneumonia on days 1, 3, 5 and 7 upon initiation of symptoms. Concentrations of tumour necrosis factor-alpha (TNFα), interleukin (IL)-6, IL-8 and G-CSF were estimated by ELISA. CD34/CD45 cells were determined after incubation with anti-CD45 FITC and anti-CD34 PE monocloncal antibodies and flow cytometric analysis. Samples from eight healthy volunteers served as controls.

**Results:**

Median of CD34/CD45 absolute count of controls was 1.0/μl. Respective values of the total study population were 123.4, 112.4, 121.5 and 120.9/μl on days 1, 3, 5 and 7 (p < 0.0001 compared to controls). Positive correlations were found between the absolute CD34/CD45 count and the absolute monocyte count on days 1, 5 and 7. Survival was prolonged among patients with less than 310/μl CD34/CD45 cells on day 1 compared to those with more than 310/μl of CD34/CD45 cells (p: 0.022). Hazard ratio for death due to sepsis was 5.47 (p: 0.039) for CD34/CD45 cells more than 310/μl. Median IL-6 on day 1 was 56.78 and 233.85 pg/ml respectively for patients with less than 310/μl and more than 310/μl CD34/CD45 cells (p: 0.021).

**Conclusion:**

Stem cells are increased in peripheral blood over all days of follow-up compared to healthy volunteers. Patients with counts on day 1 less than 310/μl are accompanied by increased survival compared to patients with more than 310/μl.

## Background

Sepsis syndrome is one of the leading causes of death occurring in more than 1,500,000 patients each year in either Europe or North America with a mortality rate ranging between 35 and 50% [1]. Current theory for pathogenesis is based on the production of pro-inflammatory mediators like tumour necrosis factor-alpha (TNFα) and interleukins (IL)-6 and IL-8 by cells of the innate immune system when triggered by constituents of bacterial cells of the responsible pathogens [2]. These cells of the innate immune system involved in pathogenesis are myeloid cells, mainly neutrophils and monocytes, derived from progenitor stem cells. During evolution of the septic process, increased apoptosis of monocytes and lymphocytes is observed [3,4], leading to hypothesis that stem cells are activated through hemopoetic mechanisms in order to replace them. Despite the importance of stem cells for maturation into neutrophils and monocytes, no data are available in the current literature about the kinetics of stem cells in the peripheral blood of the septic host. Stem cells are recognized by the expression of the CD34 surface molecule on their cell membrane [5].

The present study aimed to provide information about stem cells circulating in blood in sepsis. In an attempt to apply a study population as homogenous as possible, only patients with ventilator-associated pneumonia and sepsis were enrolled in order to minimize confounding variables occurring when patients with different underlying infections offering a great variety of antigenic stimuli for the innate immune system were encountered together.

## Methods

### Study design

A total of 44 patients were enrolled in a prospective study that took place over the period May-November 2005. The study population was different than the one described recently [4]. Patients were hospitalized in the Department of Critical Care of the "Evangelismos" General Hospital and in the 2^nd ^Department of Critical Care of the "ATTIKON" University Hospital of Athens. The study was approved by the Ethics Committee of both hospitals. All enrolled patients were intubated for at least 48 hours prior enrolment and they were aged above 18 years. Written informed consent was provided by their relatives.

Exclusion criteria were:

• any hematologic or solid tumor malignancy;

• any administration of G-CSF or CM-CSF over the last three months;

• neutropenia (≤500 neutrophils/mm^3^);

• HIV infection; and

• oral intake of corticosteroids at a dose equal to or higher than 1 mg/kg equivalent of prednisone for a period greater than one month.

Inclusion criteria were the concomitant presence of:

• ventilator-associated pneumonia (VAP), and

• sepsis, severe sepsis or septic shock.

Death due to sepsis was the clinical endpoint of the study.

### Definitions

Diagnosis of VAP was established in any patient presenting with the following signs:

• core temperature >38°C or <36°C;

• new or persistent consolidation in lung X-ray;

• purulent trancheobronchial secretions (TBS);

• clinical pulmonary infection score (CPIS) more than 6 [6-9].

CPIS was determined after individual scoring for each of the following parameters [10], as follows:

• Core temperature 36.5–38.4°C: 0 points; 38.5–38.9°C: 1 point; ≤36°C or ≥39°C: 2 points

• White blood cells 4,000–11,000/μl: 0 points; <4,000 or >11,000/μl: 1 point; >11,000 points and more than 10% bands: 2 points

• pO_2_/FiO_2 _≥240 or presence of ARDS: 0 points; pO_2_/FiO_2 _<240 in the absence of ARDS: 2 points

• Diffuse shadows on lung X-ray: 1 point; localized shadow on lung X-ray: 2 points

• Purulent TBS: 2 points

• TBS cultures yielding a pathogen ≥10^6 ^cfu/ml with negative Gram stain: 1 point; TBS cultures yielding a pathogen ≥10^6 ^cfu/ml with positive Gram stain: 2 points

Diagnosis of sepsis was based on the presence of at least two of the following [11]:

• core temperature >38°C or <36°C;

• P_co2_<32 mmHg;

• pulse rate >90/min; and

• white blood cells >12,000/μl or <4,000/μl or >10% bands.

Severe sepsis was determined as the acute dysfunction of at least one organ i.e. the acute presentation of at least one of the following [11]:

• Acute Respiratory Distress Syndrome (ARDS), as any value of pO_2_/FiO_2 _below 200

• Acute renal failure, as the production of less than 0.5 ml/Kg body weight/h of urine for at least two hours provided that the negative fluid balance of the patient was corrected

• Metabolic acidosis as any pH<7.30 or any base deficit greater than 5 mEq/l and serum lactate at least more than 2× normal value

• Acute coagulopathy as any platelet count <100.000/μl or INR> 1.5

Septic shock was considered as any value of systolic pressure below 90 mmHg requiring the administration of inotropic agents after adequate fluid resuscitation [11].

### Sample collection

Upon enrolment in the study, quantitative cultures of tracheobronchial secretions (TBS) were performed; TBS were collected after insertion of a sterile catheter in the intubation tube or in the tracheotomy connected to a negative pressure device. Enrolled patients were followed-up on a daily basis for a total of 28 days; evaluation comprised lung X-rays, estimation of the pO_2_/FiO_2 _ratio and of the APACHE II and SOFA scores.

For the estimation of cytokines and of the percentage of CD34/CD45 cells, 5 ml of blood were sampled after venipuncture of a peripheral vein under sterile conditions on the first, third, fifth and seventh days. Blood was collected into sterile tubes for the estimation of cytokines and into EDTA-coated tubes (Vacutainer, Becton Dickinson, Cockeysville MD) for the determination of CD34/CD45 cells. After centrifugation, serum was kept at -70°C until assayed.

### Laboratory techniques

#### Cultures of TBS

Quantitative TBS cultures were performed immediately after collection; 0.5 ml of sample were added into a sterile tube with 0.5 ml of 1 mg/ml of dithiothreitol (Oxoid Ltd, London, UK) and diluted five consecutive times 1:10. Volumes of 0.1 ml of each dilution were plated onto McConkey and blood agar (Becton Dickinson, Cockeysville, Md). Dishes were incubated for five days at 35°C and their count was estimated after multiplying with the appropriate dilution factor. Cultures yielding a pathogen at a count ≥1 × 10^6 ^cfu/ml were considered positive [10]. Identification of pathogens was performed by the API20E and the API20NE systems (bioMérieux, Paris L'Etoile, France).

#### Serum cytokine measurements

Concentrations of tumour necrosis factor-alpha (TNFα), interleukin-6 (IL-6), and IL-8 in sera were estimated in duplicate by an enzyme immunoabsorbent assay (Diaclone, Paris, France). Lowest limits of detection were 0.5 pg/ml for TNFα, 6.25 pg/ml for IL-6 and 62.5 pg/ml for IL-8. Concentrations of G-CSF (Granulocyte Colony Stimulating Factor) were estimated in sera of days 1 and 3 by an enzyme immunoabsorbent assay (R&D Systems, Minneapolis, USA). Lowest limit of detection was 1.25 pg/ml.

#### Flow cytometric analysis of hemopoetic cells

For the estimation of the percentage of CD34/CD45 cells, whole blood was applied. Red blood cells were lysed by the application of NH_4_Cl 0.1 M and white blood cells were washed three times with PBS pH: 7.2. Subsequently, they were incubated for 15 minutes in the dark with the monoclonal antibodies anti-CD45 FITC (Immuotech, Marseille, France) and anti-CD34 PE (Immunotech). Cells staining positive for both CD34 and CD45 after analysis by the EPICS XL/MSL flow cytometer (Beckman Coulter Co) with gating for the whole cell population, were considered hemopoetic stem cells [2]. IgG FITC and IgG PE antibodies were applied as negative controls for each sample. Mean fluorescence intensity (MFI) was also recorded. In order to evaluate the percentage of CD34/CD45 cells of the enrolled patient population, eight samples derived from healthy volunteers well matched for age and sex to the study population were applied. The absolute count of CD34/CD45 cells was estimated after multiplication of their percentage to the absolute white blood cell count determined after analysis by an automatic counter (Beckman Coulter Co).

### Statistical analysis

Results were expressed as medians ± standard errors (SE) or 95% confidence intervals (CI). Comparisons between patients and healthy controls were performed by the Mann-Whitney U test. Statistical correlations were performed after assessment of the non-parametric co-efficient of Spearman (r_s_). Survival Kaplan-Meier analysis was performed separately for patients with CD34/CD45 cells less than 310/μl and equal to or more than 310/μl. The latter threshold was applied after scatterploting of single values of survivors and non-survivors. Survival curves were compared by the log-rank test. Cox regression analysis for estimation of hazard risk (HR) and their 95%CI for death due to sepsis was performed; APACHE II score of day 1 more than 20, category of critical illness and the count of CD34/CD45 cells on day 1 were covariates. Changes of CD34/CD45 counts were estimated between days 1 and 3, between days 1 and 5 and between days 1 and 7. These changes were compared for patients with CD34/CD45 cells less than 310/μl and equal to or more than 310/μl on day 1 by the Mann-Whitney U test. Any value of p below 0.05 was considered statistically significant.

## Results

**Figure 1:**
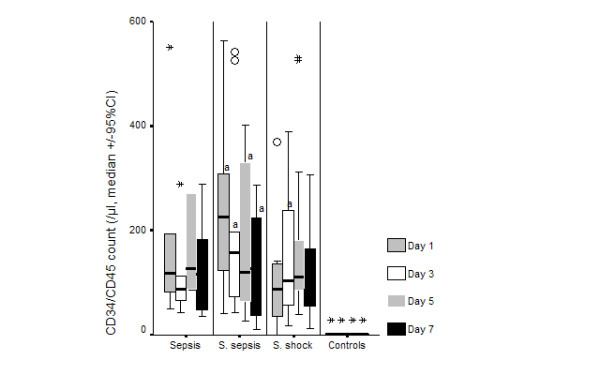
Absolute blood counts of CD34/CD45 cells over daily follow-up of patients with ventilator-associated pneumonia (VAP) and sepsis, severe sepsis or septic shock. Circles denote outliers and asterisks denote extremes. ^a^p < 0.05 compared to sepsis.

Clinical characteristics of patients enrolled in the study are shown in Table 1. Median ± SE of CD34/CD45 cells of controls was 1.0 ± 3.2/μl. Respective values of the total study population were 123.4 ± 61.6, 112.4 ± 96.8, 121.5 ± 96.8 and 120.9 ± 30.6/μl on days 1, 3, 5 and 7 of follow-up respectively (p of comparisons of all days to controls less than 0.0001). In these days median ± SE of MFI were 6.05 ± 0.33, 6.55 ± 0.33, 6.10 ± 0.58 and 6.15 ± 0.55 respectively. Follow-up values of CD34/CD45 cells separately for patients with sepsis, severe sepsis and septic shock are shown in Figure 1.

**Table 1:** Demographic characteristics of 44 patients with ventilator-associated pneumonia (VAP) and sepsis enrolled in the study.

Age (years, mean ± SD)	59.56 ± 18.28
Male/Female	32/12
APACHE II score (mean ± SD)	16.49 ± 5.52
SOFA score (mean ± SD)	8.14 ± 3.39
White blood cells (mean ± SD, /μl)	12,939.8 ± 6,502.5
Sepsis [No of patients (%)]	10 (22.72)
Severe Sepsis [No of patients (%)]	12 (27.27)
Septic shock [No of patients (%)]	22 (50.00)

Underlying conditions [No (%)]	
Multiple injuries	10 (22.72)
Chronic obstructive pulmonary disease	22 (50.00)
Celiac aortic aneurysm replacement	5 (11.36)
Others	5 (11.36)

Predisposing factors [No (%)]	
Diabetes mellitus 2	7 (15.91)
Coronary Heart Disease	10 (22.72)
Others	5 (11.36)

Positive cultures of TBS* [No (%)]	25 (56.82)
*Acinetobacter baumannii*	15 (34.09)
*Pseudomonas aeruginosa*	7 (15.91)
Others	3 (6.82)

*Tracheobronchial secretions

Median ± SE of neutrophils of the whole study population on days 1, 3, 5 and 7 were 8,303.8 ± 1,064.8, 8,683.4 ± 1,051.4, 8,748.4 ± 1,086.6 and 7.659.4 ± 919.9/μl respectively. No correlation was found between the absolute CD34/CD45 count and the absolute neutrophil count of patients in any day of follow-up. Median ± SE of monocytes of the whole study population on days 1, 3, 5 and 7 were 630.3 ± 64.0, 585.5 ± 76.9, 632.9 ± 112.7 and 568.2 ± 99.4/μl respectively. Positive correlations were found between the absolute CD34/CD45 count and the absolute monocyte count on day 1 (r_s_: +0.342, p: 0.031), on day 5 (r_s_: +0.402, p: 0.015) and on day 7 (r_s_: +0.359, p: 0.034).

**Figure 2:**
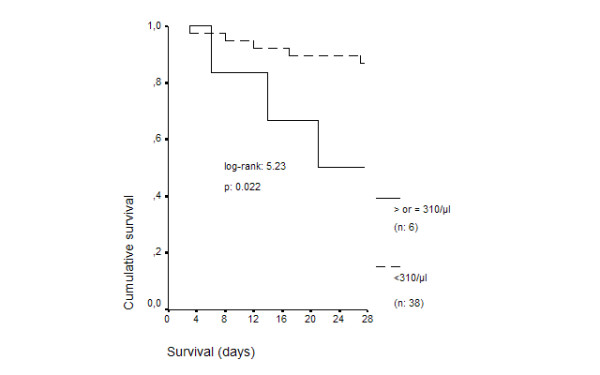
Survival curves of 44 patients with ventilator-associated pneumonia (VAP) and sepsis in relation to the absolute blood count of CD34/CD45 cells on the first day of diagnosis.

**Figure 3:**
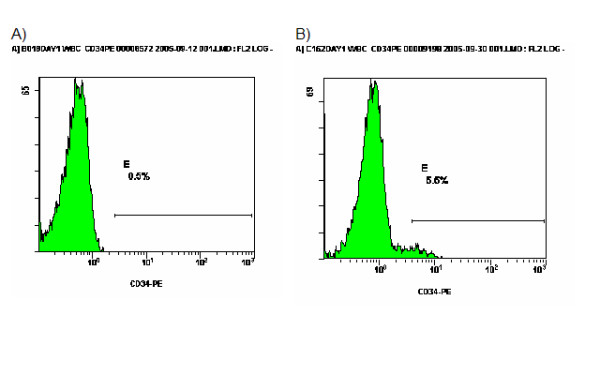
Flow plots of the expression of CD34 in two patients with ventilator-associated pneumonia (VAP) and sepsis on day 1; patient A with 180/μl CD34/CD45 cells and patient B with 1,737/μl CD34/CD45 cells.

Death from all causes supervened in 12 patients (27.27%); death attributed to the initial septic episode that lead to study enrolment occurred in eight patients (18.18%). Survival curves for patients with absolute count of CD34/CD45 cells on the first day less than 310/μl and equal to or more than 310/μl are shown in Figure 2. Survival was prolonged among patients with less than 310/μl (p: 0.022). Representative flow plots of the expression of CD34 on two patients, one with less than 310/μl CD34/CD45 cells on day 1 and another with more than 310/μl CD34/CD45 cells on day 1, are given in Figure 3.

Median ± SE changes of CD34/CD45 counts between days 1 and 3 were -2.7 ± 25.8 and +260.4 ± 284.8/μl for patients with CD34/CD45 cells less than 310/μl and equal to or more than 310/μl on day 1 respectively (p: 0.011). Respective values of changes between days 1 and 5 were -41.9 ± 111.4 and 231.4 ± 256.9/μl (pNS) and between day 1 and 7 12.2 ± 19.23 and 366.1 ± 311.2/μl (p: 0.004).

HR for death due to sepsis was 4.49 (pNS, 95%CI: 0.9122.108) for APACHE II more than 20, 2.19 (pNS, 95%CI: 0.50–9.57) for category of critical illness and 5.47 (p: 0.027, 95%C: 1.21–24.65) for CD34/CD45 cells ≥310/μl.

Median ± SE TNFα of the whole study population on days 1, 3, 5 and 7 were 9.12 ± 2.17, 10.02 ± 3.30, 9.724 ± 35.41 and 8.61 ± 18.92 pg/ml respectively. Median ± SE IL-6 of the whole study population on days 1, 3, 5 and 7 were 67.86 ± 19.89, 66.62 ± 14.89, 57.14 ± 14.79 and 59.15 ± 13.41 pg/ml respectively. Median ± SE IL-8 of the whole study population on days 1, 3, 5 and 7 were 112.5 ± 156.8, 62.5 ± 282.1, 275.6 ± 165.0 and 125.0 ± 96.1 pg/ml respectively. Median ± SE G-CSF of the whole study population on days 1 and 3 were 78.13 ± 53.75 and 73.21 ± 60.56 pg/ml respectively. Lack of any correlation was noted between CD34/CD45 absolute counts and serum levels of TNFα, IL-6, IL-8 and G-CSF on each day of follow-up.

Median ± SE TNFα on day 1 were 9.54 ± 2.45 and 21.33 ± 6.55 pg/ml respectively for patients with less than 310/μl and equal to or more than 310/μl CD34/CD45 cells (pNS). Respective values for IL-6 were 56.78 ± 22.16 and 233.85 ± 67.87 pg/ml (p: 0.021); for IL-8 were 71.3 ± 111. 9 and 62.5 ± 105.2 pg/ml (pNS); and for G-CSF were 78.12 ± 64.41 and 78.12 ± 71.99 pg/ml (pNS).

## Discussion

Current theories for the pathogenesis of sepsis implicate the dominant role of cells of the innate immune system i.e. monocytes and neutrophils for the biosynthesis of pro-inflammatory mediators after triggering by bacterial pathogens [2]; these mediators elicit further clinical signs of the septic host. Neutrophils and monocytes constitute myeloid cells derived from progenitor hemopoetic stem cells that are CD34/CD45-positive cells [5]. Based on the hypothesis that white blood cells die through apoptosis in sepsis [3,4], stem cells may be triggered for further expansion. Since no data are available in the current literature, the present study was assigned to the kinetics of progenitor stem cells in the peripheral circulation of septic patients.

Results revealed that the percentage of stem cells in blood of septic patients was higher than in healthy volunteers, a phenomenon observed for all the first seven days of follow-up. The latter finding was particularly pronounced in the event of severe sepsis (Figure 1).

The most important finding of the present study was the existence of a correlation between CD34/CD45 count of day 1 and the patients' outcome. Time of survival was decreased for patients with stem cells equal to or more than 310/μl in their blood compared to patients with stem cells less than 310/μl (Figure 2). That finding was further verified by Cox regression analysis revealing that presence of ≥310/μl stem cells was accompanied by a significantly increased risk of death of 5.47 times more than in patients with less than 310/μl stem cells. No data are available in the literature that may explain the described correlation between the level of stem cells in blood and time of survival. As a consequence, only hypotheses can be made. Based on the detected positive correlation between CD34/CD45 counts and monocytes counts, it might be presumed that increased progenitor cells would give rise to increased hemopoesis of monocytes and to subsequent augmented burden of pro-inflammatory mediators. It was of striking importance to show that in patients with CD34/CD45 higher than 310/μl on day 1, their counts fell on the consecutive days underlying the importance of increased hemopoesis of day 1 for the outcome of the patient.

The search for the impact of circulating pro-inflammatory cytokines on the count of stem cells, showed that circulating levels of IL-6 were higher on day 1 among patients with CD34/CD45 higher than 310/μl compared to patients with less than 310/μl. A similar difference was not detected for TNFα, IL-8 or G-CSF. The importance of IL-6 for triggering of hemopoesis on day 1 might be supported from in vitro evidence describing IL-6 as a promoter of the expansion of progenitor hemopoetic cells [13,14].

The above results are strengthened in the light of a study population being as homogenous as possible. All enrolled patients became septic by the same underlying infection i.e. VAP. That study design allowed to elaborate a similar antigenic stimulus to the immune system of all patients concerning the infective focus. Analysis revealed that patients were predominantly infected by species of *Pseudomonas aeruginosa *and by *Acinetobacter baumannii *(Table 1) so that the offered bacterial triggering to the entire population was the most homogenous possible.

## Conclusion

This is the first study in the literature, describing the kinetics of stem cells in the septic process. They are increased over all days of follow-up compared to healthy volunteers and they are positively correlated to absolute monocyte counts. Moreover their count on day 1 is correlated to time of survival since patients with less than 310/μl survived longer compared to patients with equal to or more than 310/μl. The latter data might unravel a novel pathophysiological pathway that although that may not be applied directly in clinical practice, their importance is enormous since all novel therapies for the septic host are targeting the pathogenetic cascade.

## Abbreviations

TNFα: tumour necrosis factor-alpha

IL-6: interleukin-6

IL-8: interleukin-8

G-CSF: granulocyte colony stimulating factor

## Competing interests

The author(s) declare that they have no competing interests.

## Authors' contributions

TT participated in the design of the study, in the estimation of inflammatory parameters and drafted the manuscript.

EJGB participated in the design of the study and drafted the manuscript.

SK participated in the enrolment of patients.

DZ participated in the enrolment of patients.

VK participated in flow cytometric analysis

AP participated in the estimation of G-CSF

ET participated in performance of total white cell counts and cytokines

MK participated in the enrolment of patients.

AK participated in the enrolment of patients.

ND drafted the manuscript

AA drafted the manuscript

CR drafted the manuscript

HG participated in study design and drafted the manuscript

All authors have read and approved the final manuscript

## Pre-publication history

The pre-publication history for this paper can be accessed here:


